# Prior intake of Brazil nuts attenuates renal injury induced by ischemia and reperfusion

**DOI:** 10.1590/1678-46a85-JBN-3819

**Published:** 2018-04-19

**Authors:** Natassia Alberici Anselmo, Leticia Colombo Paskakulis, Renata Correia Garcias, Fernanda Fortuci Resende Botelho, Giovana Queda Toledo, Maria Fernanda Ribeiro Cury, Natiele Zanardo Carvalho, Glória Elisa Florido Mendes, Tatiane Iembo, Thaís Santana Gastardelo Bizotto, Patricia Maluf Cury, Agnaldo Bruno Chies, Carla Patrícia Carlos

**Affiliations:** 1 Faculdade de Medicina de São José do Rio Preto - FACERES, Laboratório de Pesquisa Experimental, São José do Rio Preto, SP, Brasil.; 2 Faculdade de Medicina de Marília, Laboratório de Farmacologia, Marília, SP, Brasil.

**Keywords:** Ischemia, Reperfusion injury, Acute kidney injury, Oxidative stress, Inflammation, Brazil nuts, Rats, Isquemia, Reperfusão, Lesão renal aguda, Estresse oxidativo, Inflamação, Castanha-do-brasil, Ratos

## Abstract

**Introduction:**

Ischemia-reperfusion (IR) injury results from inflammation and oxidative stress, among other factors. Because of its anti-inflammatory and antioxidant properties, the Brazil nut (BN) might attenuate IR renal injury.

**Objective:**

The aim of the present study was to investigate whether the intake of BN prevents or reduces IR kidney injury and inflammation, improving renal function and decreasing oxidative stress.

**Methods:**

Male Wistar rats were distributed into six groups (N=6/group): SHAM (control), SHAM treated with 75 or 150 mg of BN, IR, and IR treated with 75 or 150 mg of BN. The IR procedure consisted of right nephrectomy and occlusion of the left renal artery with a non-traumatic vascular clamp for 30 min. BN was given daily and individually for 7 days before surgery (SHAM or IR) and maintained until animal sacrifice (48h after surgery). We evaluated the following parameters: plasma creatinine, urea, and phosphorus; proteinuria, urinary output, and creatinine clearance; plasmatic TBARS and TEAC; kidney expression of iNOS and nitrotyrosine, and macrophage influx.

**Results:**

Pre-treatment with 75 mg of BN attenuated IR-induced renal changes, with elevation of creatinine clearance and urinary output, reducing proteinuria, urea, and plasmatic phosphorus as well as reducing kidney expression of iNOS, nitrotyrosine, and macrophage influx.

**Conclusion:**

Low intake of BN prior to IR-induced kidney injury improves renal function by inhibition of macrophage infiltration and oxidative stress.

## RESUMO

**Introdução:**

a lesão por isquemia-reperfusão (IR) resulta, entre outros fatores, de inflamação e estresse oxidativo. Devido às suas propriedades anti-inflamatórias e antioxidantes, a castanha-do-brasil (BN) pode atenuar a lesão renal causada por IR.

**Objetivo:**

O objetivo foi investigar se a ingestão prévia de BN reduz a lesão e a inflamação renal causadas por IR, melhorando a função renal e o estresse oxidativo.

**Métodos:**

Ratos Wistar machos foram distribuídos em seis grupos (N=6/grupo): SHAM (controle), SHAM tratado com 75 ou 150 mg de BN, IR, e IR tratado com 75 ou 150 mg de BN. O procedimento de IR consistiu na nefrectomia à direita e oclusão da artéria renal esquerda por 30 minutos. A castanha foi administrada diariamente e individualmente por sete dias antes da cirurgia (SHAM ou IR), e mantida até o sacrifício (48h pós-cirurgia). Os seguintes parâmetros foram avaliados: creatinina, ureia e fósforo plasmáticos; proteinúria, volume urinário e depuração de creatinina; TBARS e TEAC (capacidade antioxidante) plasmáticos; expressão renal de iNOS e nitrotirosina, e influxo de macrófagos.

**Resultados:**

O pré-tratamento com 75 mg de BN atenuou os parâmetros de função renal alterados pela IR, com elevação da depuração de creatinina e o volume urinário, redução da proteinúria, ureia e fósforo plasmáticos, e diminuição da expressão de iNOS, nitrotirosina e da infiltração de macrófagos.

**Conclusão:**

A ingestão de baixa quantidade de BN, previamente ao processo de IR, melhora a função renal pela inibição da infiltração de macrófagos e do estresse oxidativo.

## INTRODUCTION

Ischemia and reperfusion inevitably occur during organ transplantation and are one of the causes of acute renal failure and graft rejection. Hypoxia causes damages in tubular cells, but the generation of reactive oxygen species (ROS) subsequent to blood reperfusion induces irreversible injury. A cascade of deleterious cellular responses leads to inflammation, cell death, and organ failure.^1^^-^ 4 Despite advances in the area, the mortality rate due to acute renal failure is high. Thus, understanding the mechanisms involved in injury caused by ischemia-reperfusion (IR) is essential to define strategies to minimize the consequences of the procedures involved in renal transplantation. Thus, we investigated the protective action of (BN), *Bertholettia excelsa*, in acute renal injury caused by IR process in rats.

An increasing number of studies have shown the beneficial action of BN in humans. This nut contains bioactive compounds such as selenium, tocopherol, phenolic compounds, folate, magnesium, potassium, calcium, protein, and mono and polyunsaturated fatty acids.^5^^-^ 6 The regular consumption of these nuts improves lipid profile and cardiovascular function, reduces oxidative stress in obese teens^7^ and in subjects with metabolic syndrome,^8^ and reduces the atherogenic risk in obese women, with increased activity of glutathione-peroxidase.^9^ The ingestion of a single portion of 20 to 50 g of BN by healthy volunteers reduced markers of inflammation, such as IL-1 (interleukin 1), IL-6, TNF-α (tumor necrosis factor alpha) and IFN-g (gamma interferon),^10^ and improved the lipid profile for a period exceeding 30 days.^11^ Also, it has been shown that the BN exerts a protective action against experimental cancer in rats.^12^^-^ 13 These studies highlight the beneficial action of BN in diseases related to oxidative stress and inflammation.

Given the increased risk of mortality due to acute renal failure and the fact that the anti-inflammatory and antioxidant activities of BN have been poorly explored in kidney injury, we evaluated these actions in an *in vivo* rat model of renal IR injury. Thus, the aim of the present study was to investigate whether the intake of nuts prevents or reduces kidney injury and inflammation, improving renal function and decreasing oxidative stress.

## METHODS

### ETHICS

All procedures performed in this study were in accordance with the ethical standards approved by the Committee for Animal Experiments and the Ethics Committee of FACERES School of Medicine (approval number 001/2015).

### ANIMALS AND PROCEDURES

Male Wistar rats (200 - 220 g) were randomly distributed into six groups (N = 6/group): SHAM (control), sham treated with 75 mg BN (SHAM + BN75), SHAM treated with 150 mg BN (SHAM + BN150), untreated IR, IR treated with 75 mg BN (IR + BN75), and IR treated with 150 mg BN (IR + BN150). The animals were housed under a 12:12 h light-dark cycle and allowed access to food and water *ad libitum*.

The IR procedure consisted of right nephrectomy and occlusion of the left renal artery with a non-traumatic vascular clamp for 30 min under anesthesia (xylazine 10 mg/Kg + ketamine 85 mg/Kg).^14^^-^ 15

BN (75 or 150 mg/animal, Belém do Pará, Brazil) were given daily and individually for 7 days before surgery (SHAM and IR), and maintained until euthanasia (48h after surgery). The doses were selected according to previous studies in humans without nephrotoxic and hepatotoxic effects.^7^^-^ 11

Animals from the SHAM groups were submitted to the same anesthesia and surgical procedures described above but without clamping the renal artery. The animals were euthanized 48 h after reperfusion with an anesthetic overdose (100 mg/Kg thiopental).

At the end of the surgery, all the animals were given 2 mg/Kg tramadol by gavage for postoperative pain control, kept in individual cages, received diet and water *ad libitum* for 48 hours. Nut intake was kept, according to the group, until the moment of sacrifice.

### RENAL FUNCTION STUDY

One day after the IR and SHAM procedures, the rats were placed in metabolic cages with urine volume collected and measured for 24 h; samples were taken at the end of this period. After euthanasia, blood samples were drawn, and renal tissue samples were collected. Creatinine and proteinuria levels were measured with a colorimetric assay in the 24-h urine as well as creatinine, urea, and phosphorus in plasma samples.

### PLASMA OXIDATIVE STRESS

The analysis of TBARS (thiobarbituric acid-reactive substances) and TEAC (Trolox equivalent antioxidant capacity) were made in plasma samples. The determination of TBARS is based on the quantification of substances that react with thiobarbituric acid, generating a color that can be detected in a spectrophotometer (535 nm, BIO-200S, Bioplus, São Paulo, SP, Brazil) and compared to the absorbance reading of the malondialdehyde standard (20 µM/L).^16^^-^ 17 TEAC was determined according to the equivalence of the potent antioxidant Trolox (6-hydroxy-2,5,7,8-tetramethylchroman-2-carboxylic acid; Sigma-Aldrich Chemical Co., 23881-3), a synthetic water-soluble analogue of vitamin E, with absorbance reading at 734 nm by spectrophotometry.^18^

### KIDNEY INJURY

For histopathology and immunohistochemistry analyses, the kidneys were fixed in a 4% paraformaldehyde in 0.1 M PBS (pH 7.4) for 24 h at 4°C, embedded in paraffin, stained with hematoxylin-eosin and evaluated on a Primo Star microscope (Carl Zeiss, Jena, Germany). Acute tubular necrosis was evaluated in the juxtamedullary region, and scores were assigned in tissue sections from the juxtamedullary segments in a blinded fashion as follows: 0 (no field affected), 1 (until 25% affected), 2 (26 to 50% affected), 3 (51 to 75% affected) and 4 (76 to 100% affected), as previously described.^14^

### KIDNEY MACROPHAGE INFILTRATION AND OXIDATIVE STRESS

To study the specific localization of macrophage, ED-1, iNOS (inducible nitric oxide synthase), and nitrotyrosine, sections of paraffin-embedded kidneys were used. This procedure was performed as previously described^19^^-^ 20 and the method consisted of an immunoperoxidase reaction. Tissue fragments were incubated overnight at 4°C with a primary monoclonal anti-iNOS Ab (1:10, sc-7271, Santa Cruz Biotechnology, CA, USA), or for 1 h at room temperature with a primary monoclonal anti-ED-1 Ab (1:1000, MCA341R, Serotec, Oxford, UK) or primary monoclonal anti-nitrotyrosine Ab (1:400, SC-32757, Santa Cruz Biotechnology, CA, USA). Quantitative analysis of ED-1 macrophages and iNOS was performed in tissue sections from the juxtamedullary segments in a blinded fashion, with counting being performed using a Primo Star microscope (Carl Zeiss, Jena, Germany). For nitrotyrosine analyses, one slide from each animal (n = 6 per group) was used, and 25 fields in the juxtamedullary region of the kidney were observed to obtain an average score, as previously described for histopathology.^19^^-^ 20

### STATISTICAL ANALYSIS

The results were first subjected to descriptive analysis and determination of normality using the Kolmogorov-Smirnov test. We applied analysis of variance (ANOVA), followed by the Newman-Keuls post-hoc test for multiple comparisons of samples with a normal distribution. The Kruskal-Wallis test followed by Dunn’s test was used for samples with a non-normal distribution. A *p* value of < 0.05 was considered significant.

## RESULTS

### BRAZIL NUTS DID NOT IMPROVE RENAL INJURY

Mild to moderate tissue injury was observed in the juxtamedullary region of kidneys from the IR groups, treated or not with 75 mg and 150 mg of BN. Acute tubular necrosis was characterized by the presence of vacuolated necrotic tubular cells and intense acidophilia, pyknosis, karyolysis, karyorrhexis, casts in tubular lumen, loss of tubular brush border, and presence of inflammatory cells. No significant difference in acute tubular necrosis scores was observed between the SHAM and IR groups (0.5 ± 0.84 SHAM *vs*. 1.71 ± 0.95 IR, 0.4 ± 0.89 SHAM + BN75, 0.83 ± 0.98 SHAM + BN150, 2.5 ± 1.38 IR + BN75 and 2.83 ± 1.17 IR + BN150; *p* > 0.05, Kruskal-Wallis test).

### BRAZIL NUTS AMELIORATED RENAL FUNCTION IMPAIRED BY IR

**Figure 1:**
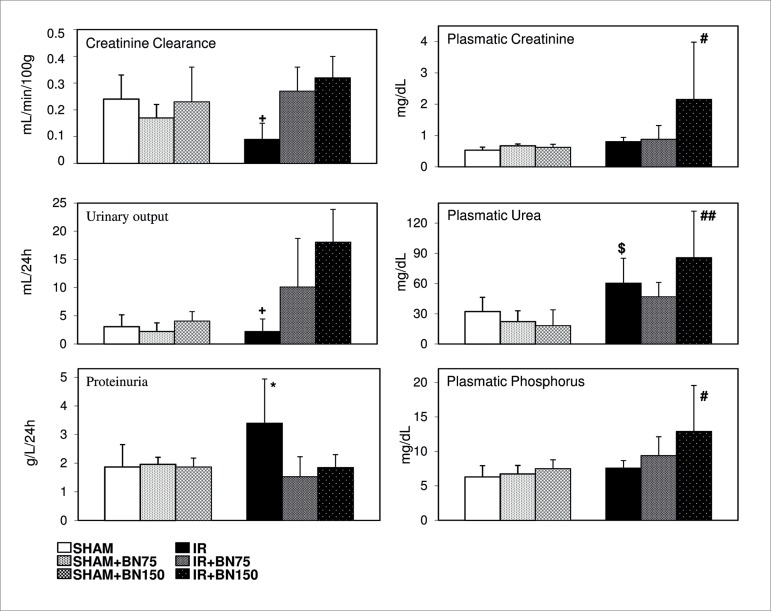
Brazil nuts ameliorated the renal function impaired by ischemia-reperfusion (IR). Kidney function decreased significantly in the group subjected to the IR process. These animals showed increased plasma urea and proteinuria and reduced urinary volume and creatinine clearance. Treatment with 75 mg of Brazil nuts (BN75) abrogated the IR effects on kidney function, compared to control groups (SHAM) treated or not with Brazil nuts and IR group. However, 150 mg of Brazil nuts (BN150) elevated plasma creatinine, urea, and phosphorus. Data are reported as mean ± standard deviation. ^*^ *p* < 0.05, IR vs. all groups; ^+^ *p* < 0.05 IR vs. IR+BN75 and IR+BN150; ^$^ *p* < 0.05 IR vs. SHAM groups; ^#^ *p* < 0.05 IR + BN150 vs. all groups; ^##^ *p* < 0.01 IR + BN150 vs. SHAM groups; n = 6/group (one-way ANOVA test).

Kidney function significantly decreased in the IR group (Figure 1). The animals showed increased plasmatic urea and proteinuria and reduced urinary volume and creatinine clearance, and these effects were abrogated by previous treatment with 75 mg BN. However, treatment with 150 mg BN elevated creatinine, urea, and phosphorus plasmatic levels compared to the other groups.

Animals from all groups lost an average of 23 g after the surgical procedures, and no difference was detected among them (data not shown).

### BRAZIL NUTS REDUCED MACROPHAGE INFLUX TO KIDNEY

**Figure 2:**
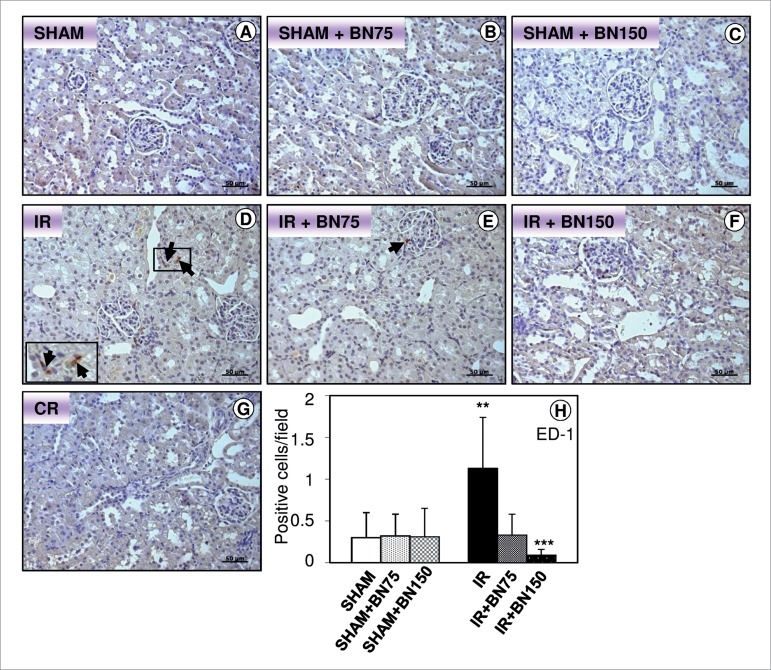
Brazil nuts reduces macrophage influx to kidney. (D) IR Group showed an increase of macrophages (arrows). There was no difference in influx of macrophages in the SHAM (A), SHAM treated with 75 mg (B) or 150 mg (C) of Brazil nuts and IR treated with 75 mg (E) or 150 mg (F) of Brazil nuts. (G) Control of reaction (CR). Counterstain: Hematoxylin. (H) Number of positive cells per field. Bars: 50 μm. Data are reported as mean ± standard deviation of protein immunoreactivity, n = 6/group. ^**^ *p* < 0.01 IR vs. all groups; ^***^ *p* < 0.001 IR + BN150 vs. IR; one-way ANOVA test.

After the IR procedure, the animals exhibited an increased influx of macrophages in the juxtamedullary region (Figures 2D-H) when compared to the other groups. A reduction of macrophage transmigration was observed in animals treated with 75 (Figure 2E and H) or 150 mg (Figure 2F and H) BN. IR animals treated with BN, either in low or in high doses, presented a similar number of macrophages compared to their SHAM controls (Figure 2A-C and H).

### BRAZIL NUTS REDUCED OXIDATIVE STRESS IN KIDNEY AND BLOOD

The IR process elevated the expression of nitrotyrosine (Figure 3.1D and H) and iNOS (Figure 3.2D and H) in kidney, as well as raised the TBARS level in blood (Figure 3.3A). Previous treatment with 75 mg BN reduced these oxidative stress markers. The dosage of 150 mg BN induced a similar effect to the low dose (Figures 3.1F and H, 3.2F and H, and 3.3A).

**Figure 3:**
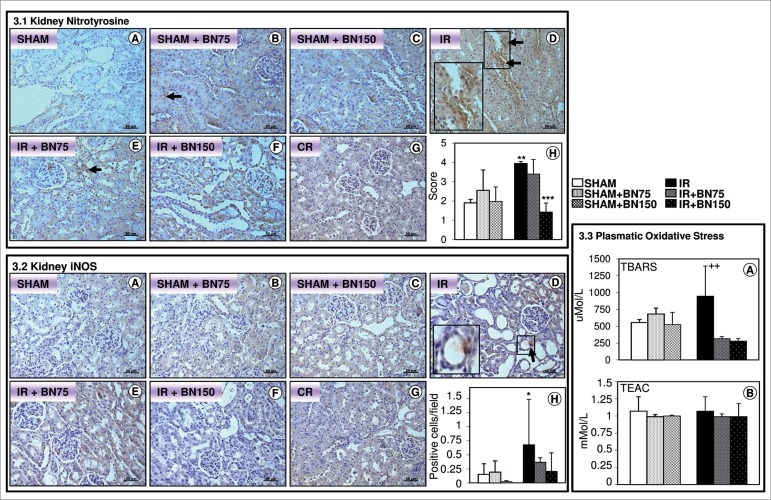
Brazil nuts reduced oxidative stress in kidney and blood. 3.1. Nitrotyrosine expression in the kidney. (D) IR Group showed increased expression of nitrotyrosine (arrows) compared to SHAM group (A). There was no difference in expression among the groups SHAM (A), SHAM treated with 75 mg (B) or with 150 mg (C), and IR treated with 75 mg (E) or with 150 mg (F) of Brazil nut. 3.2. iNOS expression in kidney. (D) IR Group showed increased expression of iNOS (arrows) compared to SHAM group treated with 150 mg of Brazil nut (C). There was no difference in expression among the groups SHAM (A), SHAM treated with 75 mg (B) or with 150 mg (C), and IR treated with 75 mg (E). 3.3. TBARS and TEAC levels in plasma. (A) IR group presented increased plasmatic TBARS (thiobarbituric acid-reactive substances), compared to IR group treated with 150 mg of Brazil nuts (IR+BN150). There were no differences among SHAM, SHAM+BN75 or SHAM+BN150 and IR treated with 75 or 150 mg of Brazil nuts. (B) The TEAC (Trolox equivalent antioxidant capacity) level was similar among all groups (p > 0.05). (G) Control of reaction (CR). Counterstain: Hematoxylin. Bars: 50 μm. (H) Average score or positive cells per field. Data are reported as mean ± standard deviation of protein immunoreactivity, n = 6/group. ^*^ *p* < 0.05 IR vs. SHAM + BN150; ^**^ *p* < 0.01 IR vs. SHAM; ^***^ *p* < 0.001 IR + BN150 vs. IR; ^++^ *p* < 0.01 IR *vs.* IR + BN150, Kruskal-Wallis test.

No significant difference in TEAC level was observed between SHAM and IR groups (Figure 3.3B).

## DISCUSSION

IR-induced renal injury causes the release of ROS and proinflammatory mediators, and the recruitment of adhesion molecules and leukocytes, which together induce kidney dysfunction and mortality.^1^^,^ 4 ^,^ 21 ^-^ 22 In the present study, an *in vivo* rat kidney IR model was used to test Brazil nuts as an agent to attenuate the IR injury in kidney.

We verified that treatment with 75 mg BN seven days before the IR process reverted the deleterious effects of IR on renal function, such as reduction of plasmatic urea and proteinuria and increase of creatinine clearance and urine volume. However, histopathology analysis revealed no difference in acute tubular necrosis among the IR groups. These results indicate a partial protective effect of BN, which is in accordance with other studies on alternative treatments for IR injury.^23^^-^ 25

In addition, the IR group exhibited elevated kidney macrophage influx, an effect abolished by both BN dosages. Macrophage cells, undetectable in normal kidneys, have consistently been used as an early renal damage indicator in humans and rodents^22^^,^ 26 ^-^ 29. Our finding thus confirms the renal injury induced by IR and the partial protective effect of BN. IR causes an early influx of macrophages to the renal tissue^1^^,^ 22 ^,^ 28 within 1 h after reperfusion followed by neutrophils,^1^ preceding a decrease in the glomerular filtration rate. This infiltration is related to an increase of chemoattractant factors.^1^^,^ 22 Different renal inflammatory processes, such as ischemia and reperfusion^22^^,^ 28 and allograft rejection,^26^^-^ 27 are characterized by an increase of chemoattractant factors including monocyte chemoattractant protein-1^27^^-^ 28 ^,^ 30 and macrophage colony stimulating factor.^29^ Monocytes cross the vascular endothelium and migrate to the damaged tissue, originating macrophages, which produce inflammatory mediators, among them, transforming growth factor (TGF-β), tumor necrosis factor (TNF-α), and interleukins 1, 6, and 12.^22^^,^ 27 ^-^ 29 ^,^ 31 The results presented demonstrate a protective effect of BN on inflammation caused by IR, with significant reduction of the transmigration of these cells in the kidneys from animals treated for 7 days before the IR surgery. This was observed with the two doses of BN (75 and 150 mg), and corroborates the anti-inflammatory action found in studies with BN consumption by healthy humans^10^ and by renal disease patients.^32^^-^ 34

In addition to the inflammatory mediators, macrophages are related to the enhancement of nitric oxide (NO), ROS, nitrotyrosine, and inflammation.^21^^,^ 30 ^,^ 35 NO may be involved in macrophage action through oxidative stress in several manifestations of nephrotoxicity. During inflammation, NO is synthesized by iNOS (inducible NO synthase) that is induced by cytokine activation, and expressed in pathological conditions; macrophages are the principal source of NO production.^36^^-^ 38 Nitrotyrosine is a result of peroxynitrite and ROS formation, and as iNOS, is a marker of nitrosative stress.^35^^,^ 39 ^-^ 41 In accordance with these authors and parallel to macrophage enhancement, our study also found elevated plasma TBARS and iNOS/nitrotyrosine renal expression caused by IR. These alterations were abolished by BN treatments and its bioactive compounds,^5^^-^ 6 which reduced macrophage influx and may have contributed to the antioxidant action. These results corroborate a study that demonstrated reduced nuclear factor kappa beta expression in peripheral blood mononuclear cells and decreased oxidative stress, cytokines, and malondialdehyde levels in hemodialysis patients that received BN supplementation.^32^^-^ 34 Moreover, the intake of BN improves glutathione peroxidase activity in obese women^9^ and adolescents^7^. Thus, our data confirms literature results about protective antioxidant effect of BN.

The renal nitrotyrosine expression in the IR+BN150 group was lower than the IR group, but not than the IR+BN75 group. The low dose of 75 mg BN might have not been enough to reduce renal expression of nitrotyrosine, although it reduced plasma level of TBARS. Also, despite the reported effect on glutathione antioxidant defense by BN^9^, we did not find improvement in TEAC plasma level of IR animals. This may be due to this treatment lasted 9 days while in other studies it was as long as 8 to 16 weeks.^7^^,^ 9

Another oxidative stress mechanism involves eNOS (endothelial NOS). The activity of eNOS is dependent on the availability of the cofactor tetrahydrobiopterin (BH4) and other cofactors.^42^ ROS indirectly affect NO bioavailability by uncoupling eNOS by oxidation of BH4. The uncoupled eNOS then produces superoxide instead of NO.^3^^,^ 42 Thus, it is proposed a suppression of eNOS by the high NO production via iNOS, which has a key role in endothelial dysfunction in acute renal ischemia. Uncoupling of eNOS and defective production of NO result in the impaired vasorelaxation in renal resistance arteries, aggravating IR injury.^43^ This ROS-mediated uncoupling mechanism of eNOS may be involved in the present model of renal injury once the expression of iNOS is elevated in IR animals. Possibly, BN administration exerts a dual protective effect (anti-inflammatory and antioxidant) by reducing macrophage infiltration with consequent reduction of iNOS expression, and helping to preserve the activity of eNOS in kidney tissue.

However, unlike treatment with 75 mg BN, kidney function of IR animals got worse with the intake of 150 mg BN. Plasma levels of creatinine, urea, and phosphorus were elevated compared to the other groups. The explanation for this deleterious effect may be the high concentrations of phosphorus and amino acids in BN.^6^ Electrolyte disorders are commonly found in kidney diseases, and nutritional support is frequently needed^44^ and low intake of protein is necessary^23^^,^ 45. Therefore, a high consumption of nuts could impair this nutritional management. In fact, the recommended amount is one nut per day by hemodialysis patients to get the anti-inflammatory and antioxidant protective effect.^32^

The present study has some limitations. The histopathological analysis showed no difference among groups, which might be due to the small number of animals; with a larger sample we might have found a significant difference as observed by other authors.^15^^,^ 28 ^-^ 29 ^,^ 36 However, the IR condition was confirmed by the elevated macrophage infiltration and decreased renal function, compared with the other groups. Our study demonstrates an interesting nutritional strategy to minimize the damaging effects of IR injury. Further studies are necessary to characterize the compounds present in BN responsible for the protective effects.

## CONCLUSION

We conclude that a low intake of BN prior to the IR-induced kidney injury improves renal function by inhibition of macrophage infiltration and oxidative stress.
